# Hospital and Health News

**Published:** 1923-04

**Authors:** 


### HOSPITAL AND HEALTH NEWS.

### A Triumph for the Schools.

The effort in the schools for the benefit of the London hospitals has produced £56,000. The London Teachers' Hospital Committee will thus have more than realised its hopes.

### Burning Household Refuse.

As a result of an appeal to Paddington householders to burn their waste paper, cardboard, vegetable matter, &c., instead of putting it into the dustbins there has been a diminution of 100 tons a month in the amount of refuse collected.

### Five Beds Founded by Working Men.

The fifth bed founded in the short period of three years by working men has been dedicated at Reigate and Redhill Hospital to the East Surrey Traction Company's Employees' Friendly Society. To name a bed an undertaking must be given to raise £70 a year.

### A New Hospital Paper.

The first number of the "Mildmay Mission Hospital Magazine" has a very attractive appearance. Issued from Austin Street, Bethnal Green, and printed on art paper, the magazine will be issued in March, June, September and December. The subscription is 1s. 6d. a year, post free.

### Premiated Designs for Chelsea.

Mr. H. V. Ashley, F.R.I.B.A., the assessor, has awarded the first prize in the competition for the design of a nurses' home at the Chelsea Hospital for Women to Messrs. Greenaway & Newberry, the second to Messrs. Horace Field & Dighton Pearson, and the third to Mr. Morley Horder. When completed the home will set free the wards now occupied by the staff, and add forty -five beds to the hospital.

### A Pamphlet on Insulin.

A pamphlet on "Insulin: a Simple Explanation of the New Treatment of Diabetes," has been issued by the Therapeutic Foods Company. (6d.) It is an attempt to explain the leading facts concerning Insulin, and a brief outline of the progress of gland therapy is also given. The little book should be of use to the layman who is anxious to know more of this treatment than can be learned from the reports in the daily newspapers.

### The Ranyard Mission.

The annual Sale of Work in aid of the Ranyard Mission was held at Ranyard House, 25 Russell Square, W.C.1, on Wednesday, March 14. There are 72 Mission Workers and 84 District Nurses living and working in the poorer districts of London, and their work is more than ever necessary in these times of difficulty and unrest. An additional income of £4,000 is necessary to maintain the Mission at its present strength, and an appeal is being made for generous support.

### A Marking Ink.

A marking ink which was the first invented, and has stood the test of a hundred years, may clearly be recommended. John Bond's "Crystal Palace" Marking Ink is of British manufacture, and has been awarded 45 Gold Medals, etc., at the International Exhibitions. It can be obtained in 6d. and 1s. bottles, either in its original form, which requires heating to fix the writing, or in the new non-heat form, which requires no heating. The ink can be obtained also by the oz., pint or quart, and is on sale at all chemists and stationers.

### A Congested Hospital.

A site for a new hospital has been bought in St. Vincent Street, to which the Glasgow Ear, Throat and Nose Hospital hopes to move. It has long been cramped in its present quarters, near by, and has occupied them since 1886. The cost of the new site is £50,000, of which nearly half is in hand. The present building was converted from domestic use; the passages are narrow, the rooms small, the staff sleep in attics, and the accommodation, in fact, is out of date.

### Health Visitors.

Mr. Charles Porter, Medical Officer of Health for Marylebone, in the course of a lecture at the Inner Temple Hall on "Principles and Practice of Sanitary Legislation," made a special claim on behalf of the health visitors, the most recent recruits to the health service. He said they should be given credit for having assisted in reducing the infant mortality rate from over 100 to under 80 per thousand births, and the average annual death rate from over 20 to round about 12 per thousand, the figure at which it stood to-day.

### Sleeping Sickness Increasing.

The Epidemiological Report of the Health Section of the League of Nations draws attention to the recrudescence of sleepy sickness (*encephalitis lethargica*) in several European countries during the last few months. There was a steady rise in the number of cases in England and Wales. During January the figures were as follows: Week ending January 6, 13; week ending January 13, 9; week ending January 20, 19; week ending January 27, 25. The number of cases for the first three weeks of February were 30, 37 and 40, thus making a total of 173 cases as against 52 cases registered during the corresponding period of 1921.

### Training Children's Nurses.

At the Princess Christian College, Wilbraham Road, Manchester, educated girls are trained as children's nurses, and babies whose parents cannot provide a home for them are received. The course of training, which lasts eight months, costs £100, including residence, board and tuition. In lieu of full fees, a student may give her services for a year and then take the course for £50. She can afterwards go for six months to the Manchester Babies' Hospital, where she is taught and maintained in return for her services.

### "The Ideal Home."

This charming monthly magazine continues to present its fortunate readers with a great variety of articles of very real interest and of hints for the improvement and adornment of the home. The March number contains eight pages of admirably reproduced photographs of Goddard's Green, Cranbrook, the picturesque retreat of Mr. E. Temple Thurston, the novelist. Clever restorations and adaptations of more or less derelict old buildings have always been a feature of "The Ideal Home," which, for the rest, deals with such varied subjects as poultry keeping and the cooking of dainty dishes. One need not be "house-proud" to appreciate its fine illustrations and wealth of practical hints.

### Retirement of Sir Frederick Mott.

Sir Frederick Mott, pathologist to the London County Mental Hospitals and Director of the Pathological Laboratory, retired from the service on March 31. He is 69 years of age, his service having been extended four years beyond the usual age for retirement. He has completed twenty-seven years of pensionable service, and the Mental Hospitals Committee are recommending the County Council to approve of six years being added to the actual term of service for the purpose of computing his superannuation allowance. They also propose that an honorarium of 250 guineas be granted to him in respect of services which he will render in an advisory capacity at the Maudsley Hospital from April 1 to October 31.

### Nurses and War Pensions.

Amended regulations under the War Pensions Act, 1921, have been issued, bringing within the scope of the War Pensions (Final Awards) Regulations, 1922, the following classes of awards, hitherto excluded: All awards in respect of disabilities attributable to or aggravated by service in the Great War notified between March 31, 1919, and August 19, 1921, in virtue of which there has been granted a gratuity, or a weekly allowance or other award (other than a wounds pension or a conditional pension), to which the previous regulations do not apply; and all awards in respect of disabilities attributable to or aggravated by service in the Great War, in virtue of which a permanent pension (other than a wounds pension) or a gratuity has been granted. Nurses who have had awards of either of these classes notified to them are entitled, if dissatisfied with their award, to appeal to the Pensions Appeal Tribunal, provided they give notice of appeal within the year ending February 6, 1924. The Pensions Appeal Tribunal is empowered to confirm, increase, decrease or set aside a final award. For further information nurses should apply to the Secretary, Ministry of Pensions, Officers Branch, 3, Sanctuary Buildings, Westminster, S.W.1.

### King Edward's Hospital Fund for London.

Hospitals in the County of London or within 9 miles of Charing Cross desiring to participate in the grants made by this Fund for the year 1923 must make application before the 31st instant to the Honorary Secretaries, 7 Walbrook, E.C.4. Applications will also be considered from convalescent homes which are situated within the same boundaries or which, being situated outside, take a large proportion of patients from London.

### Oil for Leprosy.

A despatch from Brisbane states that four women patients and one male patient, all whites, have been released from Peel Island lazarette, after satisfactorily completing their cures. Another woman patient (coloured) will soon be released. All have been successfully treated with chaulmoogra oil and its derivatives. Each released patient will be required to report at least four times in the first year of their freedom, twice in the second year, and once a year thereafter. None of them was released until after exhaustive tests extending over a considerable period.

### A Septic Ligature.

Since Lister re-introduced catgut into surgical practice much thought and labour have been expended in attempts to produce a product of unvarying sterility. The London Hospital especially has given close attention to the subject, and this study has led to the conclusion that the perfect ligature can only be obtained by beginning antiseptic preparation immediately the intestine is removed from the animal, and, further, that the separation of the mucous layer from the sub-mucous coat of the intestine must also be carried out under completely aseptic conditions. It is believed that the London Hospital catgut—sole distributors Messrs. Allen & Hanbury—satifies all the requirements of the perfect surgical ligature. This catgut is treated by a new process (Morley's), is guaranteed aseptic, and is strong and supple.

### The Rockefeller Foundation and the School of Hygiene.

The further steps necessary in regard to the formation of the School of Hygiene have been discussed between representatives of the Ministry of Health, the University of London and the Rockefeller Foundation. It has been decided to set up at once a Transitional Executive Committee to arrange for co-ordination with other authorities presently operating in the same or similar spheres; to evolve the organisation of the School of Hygiene in detail; and to develop detailed plans of the School of Hygiene to such a point that construction can actually be begun by them. It is also proposed to appoint provisionally a Director of the school who will act in an advisory capacity to the Transitional Executive Committee, and the Foundation have undertaken to provide a sum not exceeding £4,000 per annum towards the salary and expenses of the Director. The Minister of Health has under consideration the question of the personnel of the Transitional Executive Committee.

### In Aid of the Royal Northern Hospital.

Mrs. Philip Snowden, who since the Armistice has visited nearly all the European countries that were engaged in the war, recently gave a lecture on "Bolshevist Russia" at the Home and Colonial College, Wood Green. The lecture was one of a series arranged by the North London Lecture League, which is a source of income to the Royal Northern Hospital. Mrs. Snowden described the Bolshevism she found in Russia as "the same old system with a new kind of Czar," with no freedom of thought or speech. At the time of her visit—three years ago—Petrograd was a dying city, with somewhat the atmosphere of a graveyard; food was inadequate, the population dying and the hospitals in a terrible state. They had no drugs, medicines or appliances, and new born babes, lacking anything in the way of clothing, were wrapped in newspapers.—The Board of Management are opening a Neurological Section of the Out-Patient Department on April 20, and the session will be held thereafter every Friday at 1 p.m.

### World Care of Mothers.

The application of world rules for the care of mothers who work for their living is authorised in Spain by an Act which gives power to proceed with the ratification of the "Maternity Convention" adopted by the First International Labour Conference held at Washington in 1919. Under the terms of this Convention it is provided that a woman in any public or private industrial and commercial undertaking shall not be permitted to work during the six weeks following confinement and shall have the right to leave her work six weeks beforehand on production of a medical certificate. During this time she will be paid benefits sufficient for the full and healthy maintenance of herself and her child, provided either out of public funds or by means of a system of insurance and, as an additional benefit, will be entitled to free medical attendance. The "Maternity Convention" has already been ratified by Italy, Bulgaria, Greece and Roumania.

